# Supplementary material for: Using Pharmacokinetic–Pharmacodynamic Modeling to Study the Main Active Substances of the Anticancer Effect in Mice from Panax ginseng–Ophiopogon japonicus
Source: Molecules. 2024 Jan 9;29(2):334. doi: 10.3390/molecules29020334 (PMC10819458; doi:10.3390/molecules29020334)
Supplement: Supplementary file 1 [file molecules-29-00334-s001.zip › molecules-2770904-supplementary.pdf]

Using Pharmacokinetic–Pharmacodynamic Modeling to Study the Main Active Substances of the Anticancer Effect in Mice from *Panax ginseng*–*Ophiopogon japonicus*

Lu Liu [1,2], Jing Lyu[1,3,4], Longfei Yang[1,3,4], Yan Gao [1,3,4] * and Bonian Zhao [1,3,4] *

1 Institute of Pharmaceutical Research, Shandong University of Traditional Chinese Medicine,
Jinan 250355, China; 2021100041@sdutcm.edu.cn (L.L.); 60230015@sdutcm.edu.cn (J.L.); 60230001@sdutcm.edu.cn (L.Y.)

2 Innovation Research Institute of Traditional Chinese Medicine, Shandong University of Traditional Chinese Medicine, Jinan 250355, China

3 Collaborative Innovation Center for Ecological Protection and High Quality Development of Characteristic Traditional Chinese Medicine in the Yellow River Basin, Jinan 250355, China

4 High Level Traditional Chinese Medicine Key Disciplines of the State Administration of Traditional Chinese Medicine, Pharmaceutics of Traditional Chinese Medicine, Jinan 250355, China

* Correspondence: 60230003@sdutcm.edu.cn (Y.G.); 60011983@sdutcm.edu.cn (B.Z.)

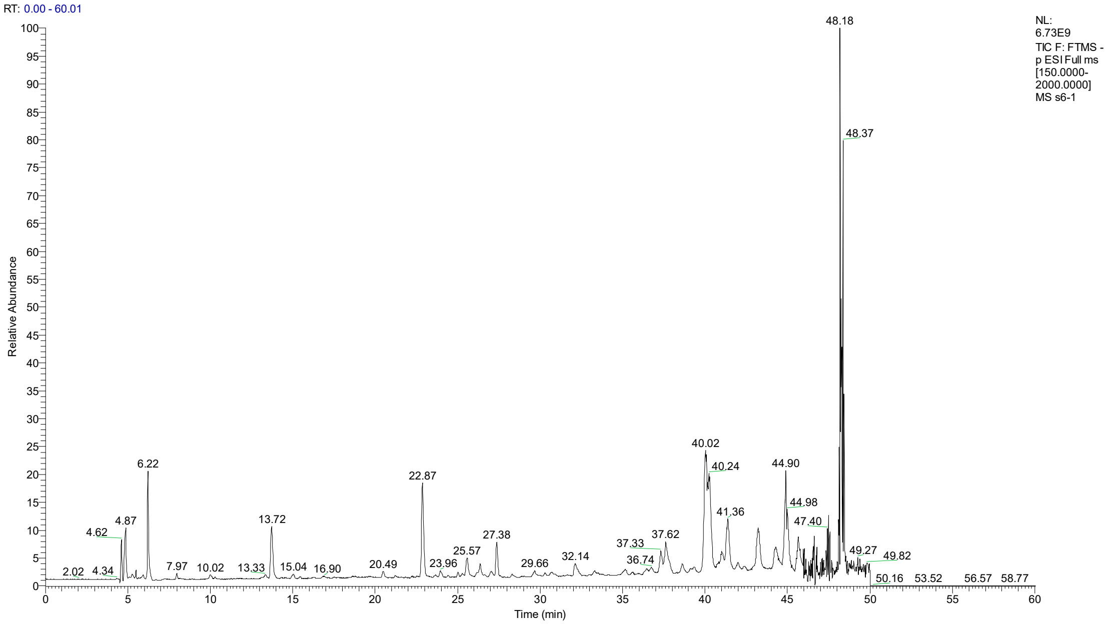

Figure S1. Total ion chromatogram of lung tissue samples in negative ion mode.

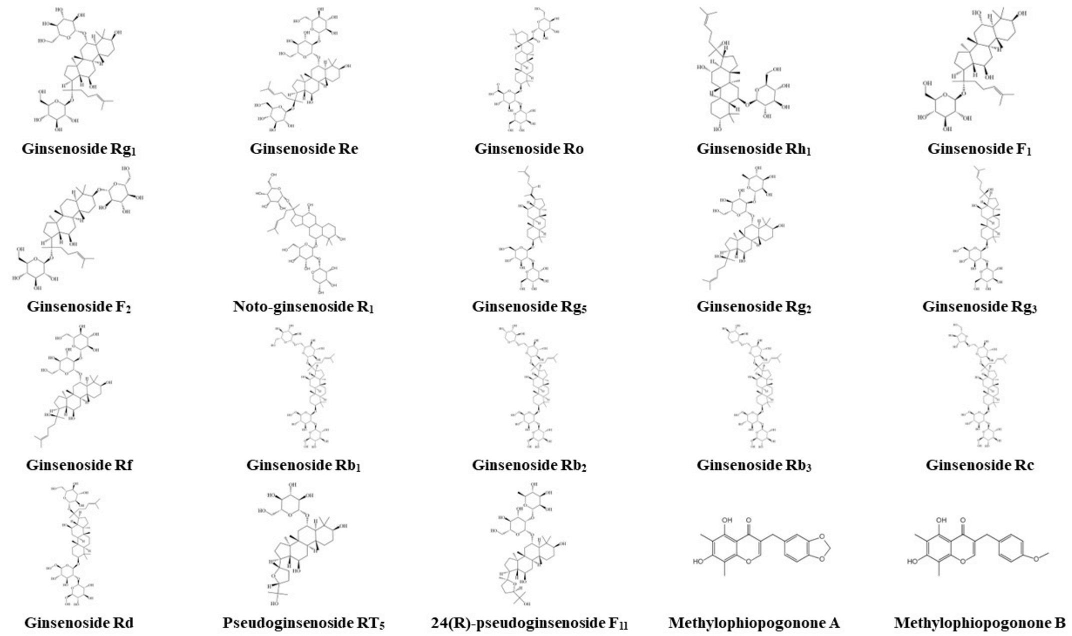

Figure S2. Chemical structures of the 20 compounds.

Table S1. The comprehensive weighted scoring of drug efficacy indicators.

Time (h)	IFN-γ	TNF-α	TGF-β1	Combined Weighted Score (S)
0.083	0.9418	0.9379	0.2634	0.1389
0.167	0.1200	1.3515	0.6516	0.1924
0.25	0.3715	0.4455	0.4645	0.0651
0.5	0.3134	0.3145	1.3691	0.0810
0.75	0.7140	0.2269	1.5642	0.0958
1	0.6402	0.0542	0.7059	0.0491
2	0.8941	0.1797	1.5414	0.0874
3	1.2556	0.9594	2.2131	0.2158
4	0.3165	0.1596	0.7663	0.0458
6	0.2799	0.0593	0.7245	0.0194
8	0.0361	0.0351	0.4682	0.0095
12	0.0378	0.0369	0.4742	0.0154
24	0.0385	0.04055	0.5016	0..0308

Table S2-1. Detection results of drug-containing lung tissue content at different time points (mean ± SD, n =6).

**Time (h)**	Ginsenoside Rg_1 (ng/g)	Ginsenoside Re (ng/g)	Ginsenoside Ro (ng/g)	Ginsenoside Rh_1 (ng/g)	Ginsenoside F1 (ng/g)	Ginsenoside F2 (ng/g)	Notoginsenoside R1 (ng/g)	Ginsenoside Rg_5 (ng/g)	Ginsenoside Rg_2 (ng/g)	Ginsenoside Rg_3 (ng/g)
0.083	41.21±9.63	132.49±32.88	11.83±0.79	8.29±0.69	0.42±0.07	14.7±0.96	5.86±1.30	4135.21±269.18	61.52±2.18	4.61±0.20
0.167	2.8±0.1	5.33±0.56	1.69±0.13	0.05±0.04	0.05±0.03	1.84±0.3	5.16±2.87	386.08±26.98	8.29±0.36	3.45±0.09

0.25	50.24±6.67	64.38±5.85	11.32±1.58	8.53±0.99	0.57±0.08	4.89±1.3	3.51±2.35	2348.06±325.95	67.24±10.16	3.91±0.16
0.5	9.25±1.73	55±21.54	3.65±0.5	1.4±0.66	0.15±0.06	6.12±3.57	3.46±1.15	658.61±156.18	17.29±1.44	3.85±0.25
0.75	4.95±0.04	5.83±0.04	1.4±0.05	2.79±0.05	0.46±0.04	17.18±0.13	0.03±0.04	4804.2±37.85	31.91±0.04	3.86±0.04
1	3.53±0.20	3.09±0.66	3.09±0.16	3.7±0.11	2.38±0.21	135.65±2.66	0.04±0.01	5679.31±158.09	52.13±1.23	4.34±0.19
2	0.82±0.06	1.54±0.11	1.39±0.05	0.31±0.02	0.11±0.02	3.7±1.03	5.17±0.37	136.75±14.00	7.15±0.24	3.98±0.73
3	3.19±0.35	12.42±0.27	2.03±0.43	1.6±0.25	1.46±0.18	45.74±2.56	6.4±1.28	2337.69±334.68	24.2±1.49	4.46±0.03
4	8.63±0.51	3.72±0.09	2.34±0.16	7.49±0.55	4.12±0.45	128.46±8.7	5.47±0.38	7967.81±441.77	69.08±2.87	4.41±0.29
6	0.58±0.02	1.7±0.15	1.35±0.13	0.42±0.01	0.27±0.01	2.55±0.73	2.22±0.12	81.51±18.10	6.00±0.09	3.4±0.08
8	0.4±0.02	0.77±0.04	1.33±0.01	0.47±0.02	0.25±0.01	1.55±0.09	0.58±0.05	138.3±22.36	6.04±0.03	3.39±0.05
12	0±0	0±0	0±0	0±0	0±0	0±0	0±0	0±0	0±0	0±0
24	0±0	0±0	0±0	0±0	0±0	0±0	0±0	0±0	0±0	0±0

Table S2-2. Detection results of drug-containing lung tissue content at different time points (mean ± SD, n =6).

**Time (h)**	Ginsenoside Rf (ng/g)	Ginsenoside Rb_1 (ng/g)	Ginsenoside Rb_2 (ng/g)	Ginsenoside Rb_3 (ng/g)	Ginsenoside Rc (ng/g)	Ginsenoside Rd (ng/g)	Pseudoginsenoside RT_5 (ng/g)	24(R)-pseudoginsenoside F11	Methylophiopogon flavanone A	Methylophiopogon flavanone B
0.083	36.13±1.77	84.51±3.17	2.22±0.15	2.54±0.16	17.39±0.64	43.37±1.94	2.62±0.23	35.98±1.93	6.21±5.23	6.44±3.18
0.167	2.22±0.12	10.62±2.98	0.67±0.05	1.63±0.05	10.91±0.29	2.86±0.40	1.77±0.36	1.98±0.11	4.2±0.11	4.54±0.80
0.25	47.9±6.96	91.22±17.84	1.9±0.29	2.62±0.12	16.7±1.45	34.48±2.62	5.11±0.88	47.49±6.94	2.04±0.29	3.45±0.17

0.5	9.09±1.97	34.9±13.81	1.01±0.10	1.84±0.01	13.87±0.58	71.57±12.27	3.38±0.76	9.23±1.73	2.33±0.27	4.37±0.60
0.75	8.08±0.04	4.52±0.04	0.72±0.04	1.53±0.04	1.31±0.04	2.93±0.04	1.79±0.04	7.8±0.04	2.95±0.04	4.9±0.04
1	8.87±0.20	26.77±1.71	0.92±0.08	1.74±0.04	0.92±0.15	23.56±0.34	1.46±0.07	10.44±0.94	2.76±0.16	1.58±0.53
2	1.25±0.20	5.33±0.56	1.09±0.17	1.54±0.06	3.89±0.13	2.57±0.10	1.31±0.09	3.83±0.21	2.06±0.11	2.12±0.17
3	5.27±0.26	128.72±5.25	1.17±0.26	1.68±0.10	1.75±0.90	9.88±0.30	1.71±0.10	7.58±0.28	5.41±0.92	20.61±2.38
4	18.78±0.77	25.54±1.63	0.79±0.02	1.54±0.02	2.32±0.20	17.81±1.40	2.28±0.12	21.21±0.93	2.92±0.10	3.51±0.66
6	0.37±0.02	14.85±0.45	0.69±0.01	1.44±0.02	4.05±0.04	2.86±0.18	1.64±0.06	2.82±0.01	2.04±0.07	2.09±0.89
8	0.12±0.01	8.75±0.16	0.69±0.01	1.44±0.03	4.16±0.01	1.74±0.02	1.46±0.08	2.62±0.05	2.32±0.38	1.54±0.20
12	0±0	440.68±15.67	7.2±0.96	5.92±0.45	42.95±2.11	82.95±2.11	0±0	0±0	0±0	0±0
24	0±0	5.51±0.36	0.78±0.06	1.79±0.08	2.35±0.89	3.16±0.54	0±0	0±0	0±0	0±0

Table S3. Reference substance concentration gradient and QC sample concentration.

Components	Nominal Concentration (mg/g)	Concentrations of QC Samples (ng/g)		
		Low	Medium	High
Ginsenoside Rg_1	1.07	5	50	500
Ginsenoside Re	0.98	5	50	500
Ginsenoside Ro	1.06	5	50	500
Ginsenoside Rh_1	1.01	5	50	500
Ginsenoside F_1	1.11	5	50	500
Ginsenoside F_2	0.97	5	50	500
Notoginsenoside R_1	1.07	5	50	500
Ginsenoside Rg_5	1.00	5	50	500
Ginsenoside Rg_2	1.18	5	50	500

Ginsenoside Rg_3	1.12	5	50	500
Ginsenoside Rf	1.06	5	50	500
Ginsenoside Rb_1	1.03	5	50	500
Ginsenoside Rb_2	0.93	5	50	500
Ginsenoside Rb_3	1.01	5	50	500
Ginsenoside Rc	1.05	5	50	500
Ginsenoside Rd	1.04	5	50	500
Pseudoginsenoside RT_5	1.11	5	50	500
24(R)-pseudoginsenoside F_{11}	1.03	5	50	500
Methylophiopogon flavanone A	0.95	5	50	500
Methy ophiopogon flavanone B	0.97	5	50	500
